# Hematopoietic Deficiency of miR-223 Attenuates Thrombosis in Response to Photochemical Injury in Mice

**DOI:** 10.1038/s41598-017-01887-x

**Published:** 2017-05-09

**Authors:** Hui Wang, Qian Wang, Kyle Kleiman, Chiao Guo, Daniel T. Eitzman

**Affiliations:** 1 Department of Internal Medicine, Cardiovascular Research Center, University of Michigan, Ann Arbor, Michigan USA; 2 Department of Cardiology, the Fourth Affiliated Hospital of China Medical University, Shenyang, China

## Abstract

Some studies have shown that levels of MicroRNA (miR)-223 derived from platelets in the plasma are reduced following inhibition of platelet function, while others have shown a correlation between low plasma miR-223 and high on-treatment platelet reactivity. The present study seeks to investigate the role of miR-223 in arterial thrombosis. A model of photochemical-induced carotid thrombosis was applied to miR-223 deficient mice and littermate (WT) controls. Mice deficient in miR-223 exhibited significantly prolonged times to occlusive thrombosis compared to WT mice indicating a protective effect of miR-223 deficiency. Bone marrow transplantation experiments confirmed that the hematopoietic pool of miR-223 was responsible for differences in thrombosis times. Transfusion of either WT platelets or extracellular vesicles derived from WT platelets were both sufficient to shorten thrombosis times in miR-223 deficient recipients. The effect of platelet transfusions on IGF-1R was explored. These experiments revealed that vascular IGF-1R was down-regulated by platelet miR-223. Furthermore, inhibition of IGF-1R abolished the protection conferred by miR-223 deficiency on thrombosis. In conclusion, platelet miR-223 is a regulator of arterial thrombosis following endothelial injury through effects on vascular wall IGF-1R. This study indicates that platelet miR-223 is a potential therapeutic target for prevention of arterial thrombosis.

## Introduction

MicroRNA (miR)-223 has been shown to be highly expressed in platelets and may serve as a biomarker of platelet activation status^1^. Some studies have shown that levels of miR-223 in the plasma are reduced following inhibition of platelet function^1^, while others have shown a correlation between low plasma miR-223 levels and high on-treatment platelet reactivity^2, 3^. In addition to serving as a biomarker of platelet activation, miR-223 may directly affect platelet function^4^. Recently, however, miR-223 was found to have no effect on platelet activation, adhesion, or aggregation *in vitro*, as well as no effect on tail bleeding times in mice^5^. The purpose of this study was to evaluate the effect of miR-223 *in vivo* using a murine model of arterial thrombosis.

## Results and Discussion

**Figure 1:**
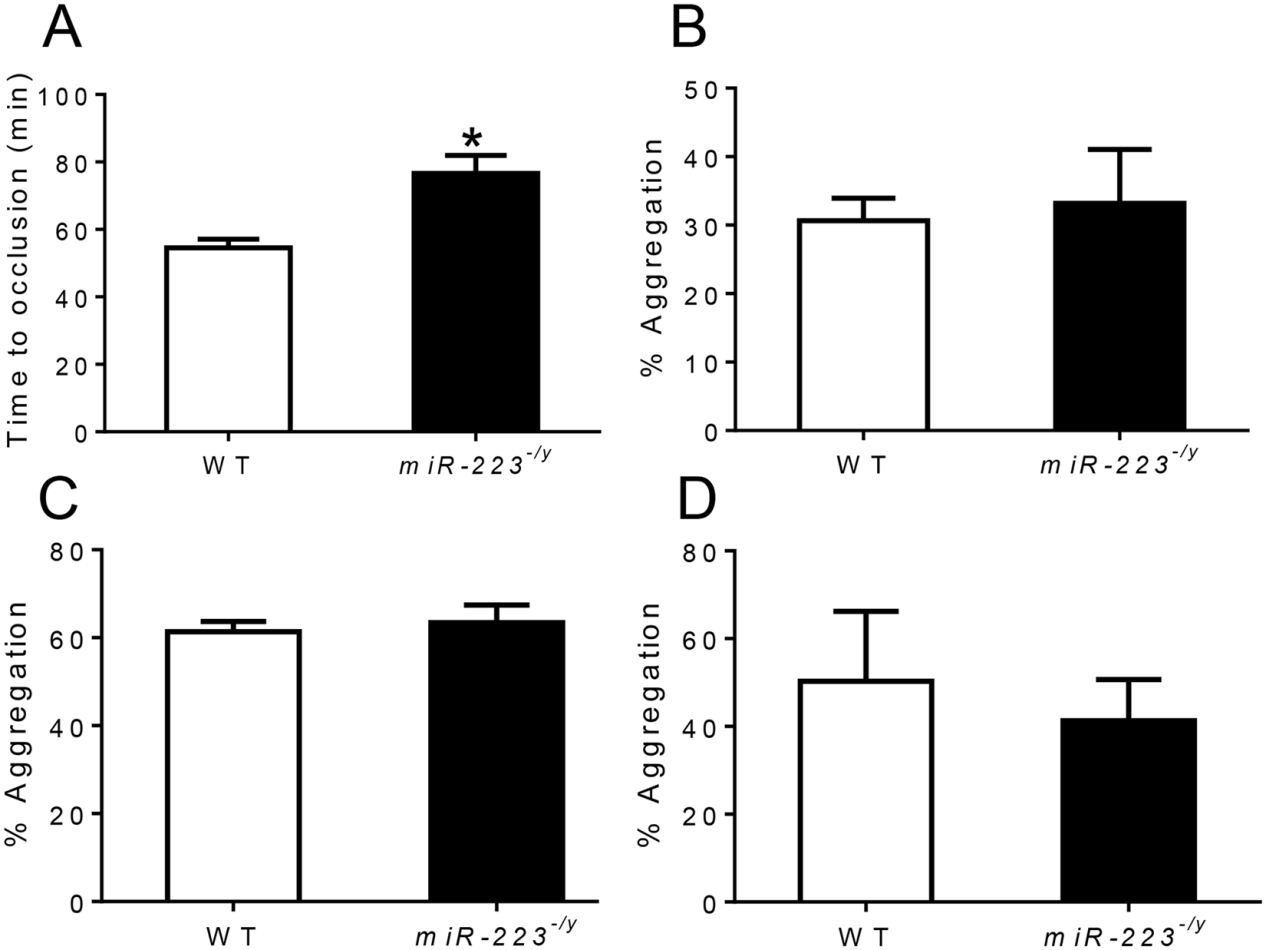
(**A**) Time to occlusive carotid thrombosis in wild-type (WT) and *miR-223* ^−/*y*^ mice (n = 10 mice per group). (**B**,**C**,**D**) Platelet aggregation of WT and *miR-223* ^−/*y*^ mice in response to ADP (**B**), collagen (**C**), and thrombin (**D**) (n = 5 mice per group). *P < 0.01 compared to WT.

To investigate the role of miR-223 on arterial thrombosis following endothelial injury, we performed rose bengal photochemical injury to the carotid arteries of WT and miR-223 deficient mice. Arterial thrombosis was defined as flow cessation for at least 10 minutes. In this model, time to occlusive thrombosis has been shown to correlate with blood platelet thrombogenicity, as well as other coagulation and fibrinolytic factor deficiency states^6^. Following carotid injury, miR-223 deficient mice exhibited significantly prolonged times to occlusive thrombosis compared to WT mice (Fig. 1A), indicating a protective effect of miR- 223 deficiency on thrombosis in this model. In this study, platelet counts were lower in miR-223 deficient mice (644.6 ± 54.2 × 10^3^/μl) compared with WT mice (901.8 ± 67.1 × 10^3^/μl) (n = 5 per group, P < 0.05). In agreement with a previous study^5^, platelet aggregation in response to ADP, collagen, or thrombin was similar between WT and miR-223 deficient mice (Fig. 1B,C,D).

**Figure 2:**
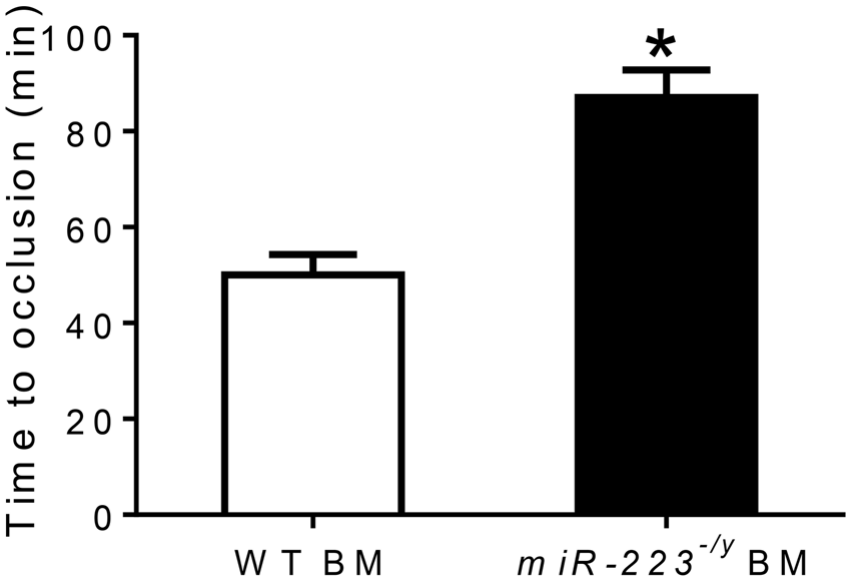
Time to occlusive carotid thrombosis in wild-type (WT) mice receiving WT bone marrow (BM) or *miR-223* ^−/*y*^ BM (n = 10 mice per group). *P < 0.01 compared to WT mice receiving WT BM.

Although miR-223 is highly expressed in platelets, other cell types also express miR-223^7^. To determine whether the hematopoietic pool of miR-223 was responsible for differences in thrombosis times, BMT was performed from miR-223 deficient mice into WT recipients, and compared to control WT recipient mice that received WT BM. 8 weeks following BMT, platelet counts were lower in mice receiving miR-223 deficient marrow compared to mice receiving WT marrow (785.2 ± 25.9 × 10^3^/μl vs 925.4 ± 21.1 × 10^3^/μl, n = 5 per group, p < 0.01), while RBC and WBC counts were similar (RBC: 9.44 ± 0.1 × 10^6^/μl vs 9.53 ± 0.1 × 10^6^/μl, n = 5 per group, p = 0.38; WBC: 9.74 ± 1.3 × 10^3^/μl vs 9.44 ± 1.1 × 10^3^/μl, n = 5 per group, p = 0.87). Following carotid photochemical injury, WT mice transplanted with WT marrow formed occlusive thrombosis with times similar to non-transplanted WT mice (Fig. 2). The time to occlusive thrombosis in WT mice receiving bone marrow from miR-223 deficient mice was markedly prolonged and similar to times observed in non-transplanted miR-223 deficient mice (Fig. 2). These data establish the hematopoietic pool of miR-223 as playing a regulatory role in carotid thrombosis following endothelial injury.

**Figure 3:**
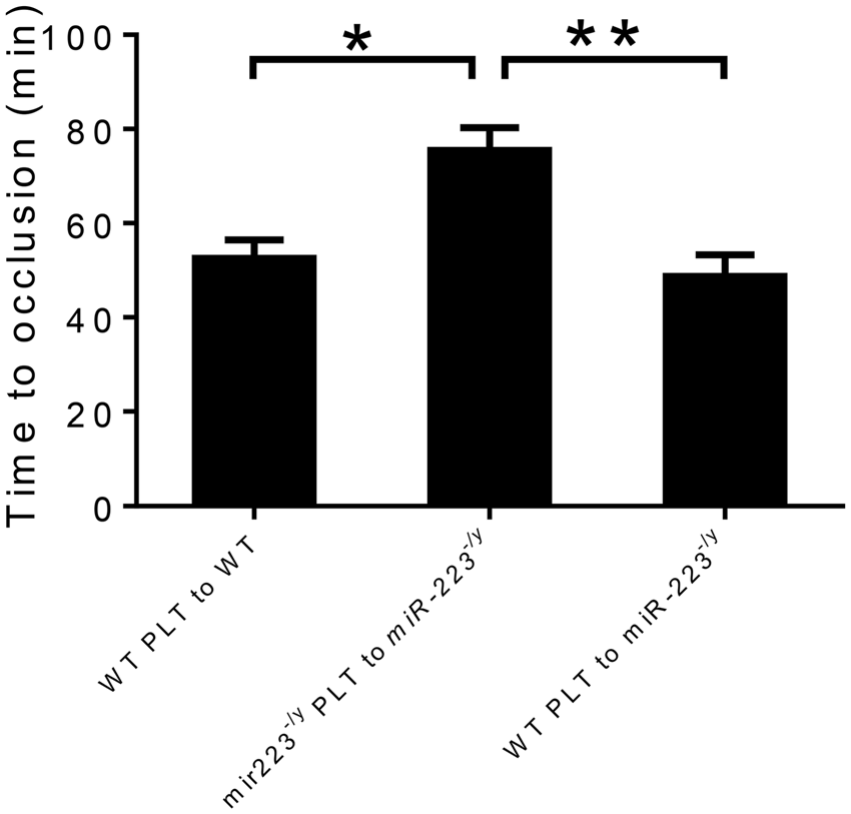
Time to occlusive carotid thrombosis in wild-type (WT) and *miR-223* ^−/*y*^ mice after platelet (PLT) transfusion (n = 10 mice per group). *P < 0.05. **P < 0.01.

To narrow down the relevant cell type even further, platelet transfusion experiments were performed in attempts to determine whether the platelet miR-223 pool was mediating the effects on thrombosis. When WT platelets were transfused into WT recipients, the time to occlusion was similar to WT mice in the absence of transfusion (Fig. 3). Similarly, when miR-223 deficient platelets were transfused into miR-223 deficient recipients, the time to thrombosis was similar to miR-223 mice in the absence of transfusion. However, when WT platelets were transfused into miR-223 deficient recipients, the protection from thrombosis was lost and thrombosis times were similar to WT mice (Fig. 3).

**Figure 4:**
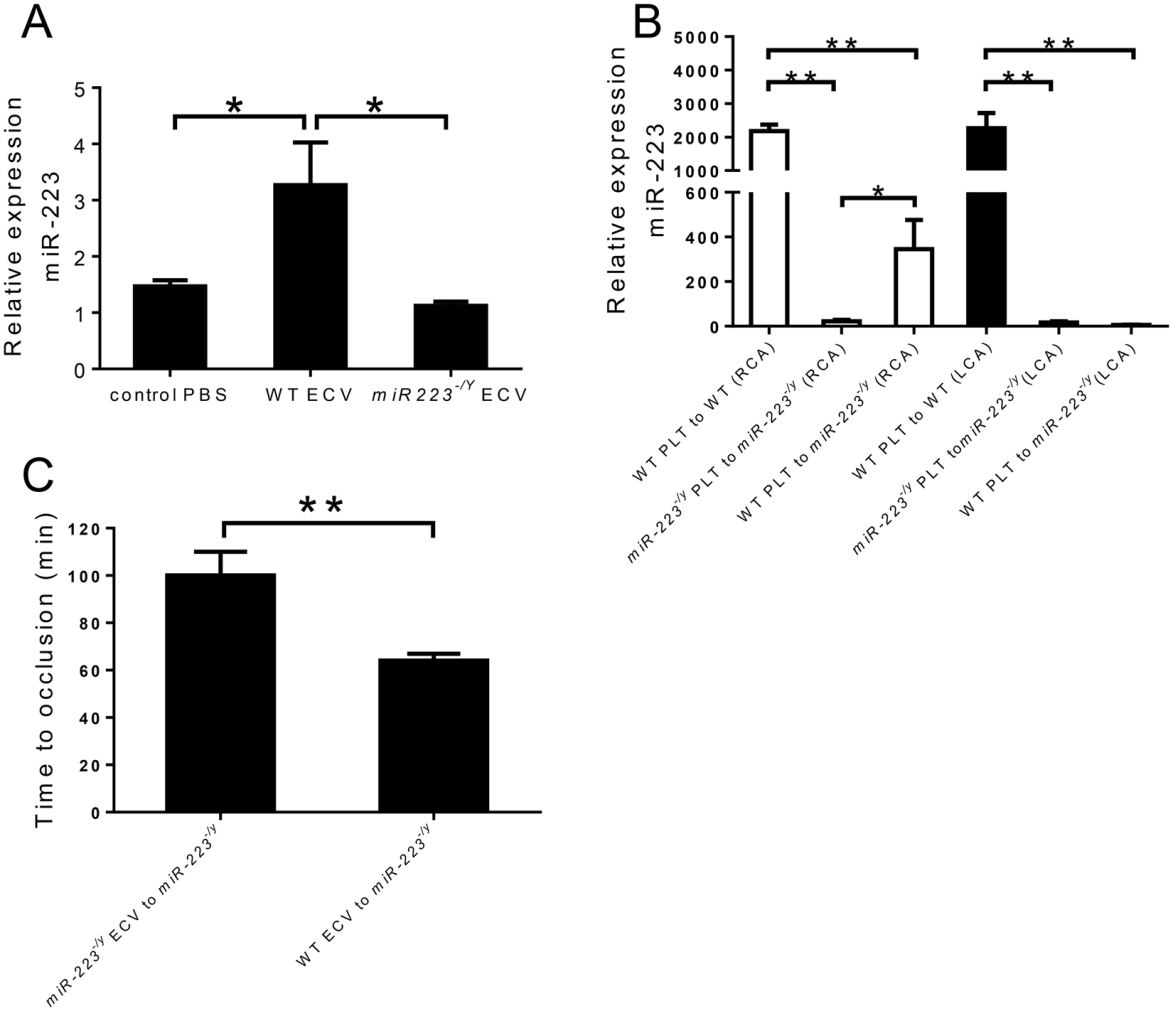
(**A**) Expression level of miR-223 in mouse brain endothelial cells (bEnd.3) after co-culture with extracellular vesicles (ECVs) from WT or *miR-223* ^−/*y*^ mice (n = 4 wells per group). (**B**) Expression level of miR-223 in left and right common carotid arteries (LCA and RCA) after platelet (PLT) transfusion and thrombosis (n = 5 mice per group). (**C**) Time to occlusive carotid thrombosis in *miR-223* ^−/*y*^ mice after WT or *miR-223* ^−/*y*^ ECV transfusion (n = 5 mice per group). *P < 0.05, **P < 0.01.

The mechanism by which miR-223 platelets affect thrombosis time is unclear. Since platelet aggregation studies were similar between *miR-223* ^−/*y*^ and WT mice, we pursued the possibility that miR-223 might be secreted from the platelet after carotid injury and affect an endothelial phenotype. For example, platelet-secreted miR-223 has been shown to be released from platelets and transported to endothelial cells^8^. Others have also shown platelet-released miR-223 promotes endothelial cell apoptosis via regulatory effects on IGF-1R^9^. To determine whether platelet miR-223 could be transferred to endothelial cells, ECVs from WT or miR-223 deficient mice were incubated overnight with bEnd3 cells. After washing bEnd3 cells and then isolating RNA, expression of miR-223 was increased in bEnd3 cells incubated with WT ECVs (Fig. 4A). To validate this platelet-to-endothelial miR-223 transfer in a more physiological setting, miR-223 expression was analyzed in the carotid arterial wall following injury from miR-223 deficient recipients that received WT platelet transfusions. Even after removal of thrombus, miR-223 could be detected from the injured artery but not the contralateral non-injured carotid artery (Fig. 4B). These findings suggest that platelets transfer miR-223 to sites of arterial injury. To determine whether ECVs isolated from platelets were sufficient to affect the thrombosis phenotype, we next examined the effect of ECV transfusion, isolated from WT or miR-223 deficient platelets, to recipient miR-223 deficient mice on arterial thrombosis. When miR-223 deficient ECVs were transfused into miR-223 deficient mice, the time to occlusion was similar to miR-223 deficient mice in the absent of transfusion. However, when WT MVs were transfused into miR-223 deficient mice, the protection conferred by miR-223 deficiency was abolished (Fig. 4C).

**Figure 5:**
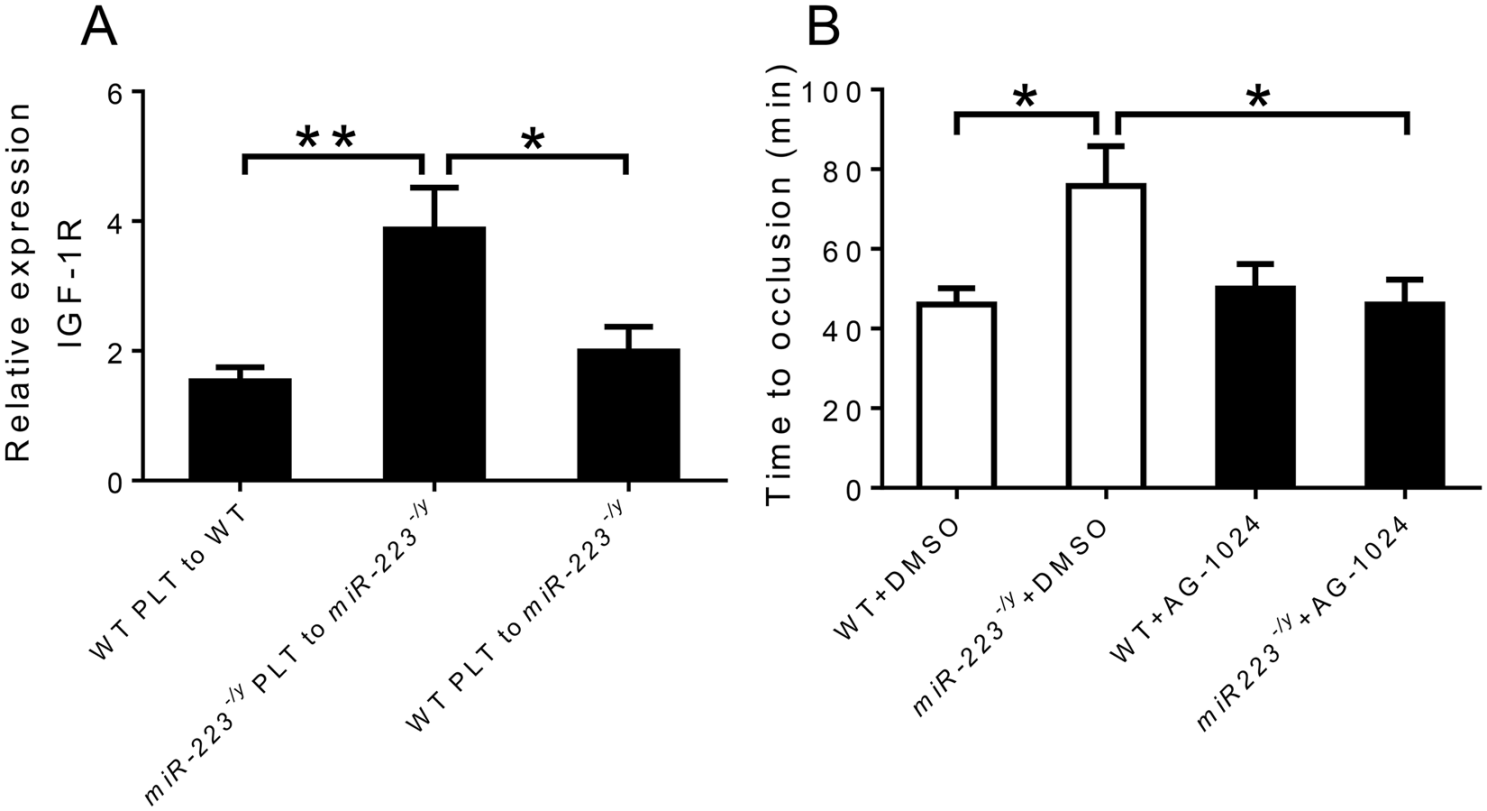
(**A**) Expression level of insulin growth factor-1 receptor (IGF-1R) in right common carotid arteries after platelet (PLT) transfusion (n = 5 mice per group). (**B**) Time to occlusive carotid thrombosis in wild-type (WT) and *miR-223* ^−/*y*^ mice after IGF-1R inhibition by AG-1024 (n = 5 mice per group). *P < 0.05. **P < 0.01.

Since IGF-1R is a miR-223 target and has shown to be involved in endothelial apoptosis^9^, we measured IGF-1R expression in injured carotid artery segments of miR-223 deficient mice that received WT or miR-223 deficient platelets. IGF-1R expression was elevated in miR-223 deficient mice that received miR-223 deficient platelet transfusions and this upregulation was abolished when WT platelets were infused into miR-223 deficient mice (Fig. 5A). Whether these effects of miR-223 on IGF-1R expression are responsible for the regulatory effects on thrombosis are not clear, however treatment of mice with an IGF-1R antagonist neutralized the protective effect of miR-223 deficiency on thrombosis (Fig. 5B). Previous clinical studies have associated low IGF-1 and IGF-1R expression in plaque tissue with clinical cardiovascular events, independent of traditional risk factors^10, 11^. Consistently, elevated IGF-1 expression is associated with attenuation of age-related endothelial progenitor cell dysfunction and senescence^12^.

MicroRNAs have been shown to be potentially important regulators of complex biological processes^13^. This study indicates that hematopoietic miR-223 is a potent regulator of arterial thrombosis following endothelial injury. This study also supports a role for cell autonomous miR-223 in platelet formation as lack of miR-223 was associated with a modest, but significant reduction in platelet counts. A recent study indicated that miR-223 deficiency is not associated with differences in platelet function^5^ and our studies confirm this. However, our *in vivo* studies do support a role for platelet miR-223 in arterial thrombosis. Consistent with previous studies that have demonstrated hematopoietic miR-223 may be transferred to other cell types such as endothelial cells^8, 9^, and affect properties of endothelial cells^14^, we demonstrated that platelet-to-endothelial transfer of miR-223 regulates thrombosis and that this effect is related to changes in vascular IGF-1R expression.

This study indicates that inhibition of platelet miR-223, or activation of its downstream target IGFR1, are potential therapeutic strategies for prevention of arterial thrombosis.

## Methods

### Animals

Male miR-223 deficient (miR-223^−/y^) mice on the C57BL6/J strain background were purchased from Jackson Laboratory (Bar Harbor, Maine) and crossed to wild-type (WT) C57BL6/J females. Heterozygous females were then crossed with WT males to generate litters in which half of the male offspring were miR-223^−/y^ and half were miR-223^+/y^ (WT). PCR primers and conditions were as described previously^7^. For all experiments, WT littermates were used as control mice. Mice were housed under specific pathogen-free conditions in static microisolator cages with tap water ad libitum in a temperature-controlled room with a 12:12-hour light/dark cycle and were fed a standard laboratory rodent diet (No. 5001, TestDiet, Richmond, IN). Complete blood counts were measured in the Unit for Laboratory Animal Medicine core at the University of Michigan using a Hemavet 950 haematology system (Drew Scientific, Miami Lakes, FL). All animal use protocols complied with the Principles of Laboratory and Animal Care established by the National Society for Medical Research and were approved by the University of Michigan Committee on Use and Care of Animals.

### Bone marrow transplantation

Bone marrow transplantation (BMT) was performed as previously described^15^. 8 week-old male WT mice were used as recipients receiving bone marrow from WT or *miR-223* ^−/*y*^ male donors. Briefly, bone marrow was harvested from the donor mice by flushing their femurs and tibias with RPMI medium (Gibco/Invitrogen, Carlsbad, CA) containing 10% fetal bovine serum (Gibco/Invitrogen, Carlsbad, CA). Cells were then centrifuged at 300 g and resuspended in phosphate-buffered saline before injection. Each recipient mouse was irradiated (2 × 650 rad [0.02 × 6.5 Gy]) and injected with 4 × 10^6^ bone marrow cells via the tail vein. Eight weeks after transplantation, blood was withdrawn from the retro-orbital sinus, and complete blood count was performed. The arterial thrombosis protocol was performed the following day.

### Carotid arterial thrombosis

To induce thrombosis, photochemical injury^16^ was performed on carotid arteries from non-BMT mice (WT vs *miR-223* ^−/*y*^) or BMT mice receiving WT or *miR-223* ^−/*y*^ bone marrow, Briefly, mice were anesthetized and secured in the supine position under a dissecting microscope (Nikon SMZ-2T, Mager Scientific, Inc., Dexter, MI). The right common carotid artery was isolated and blood flow was monitored with a doppler flow probe (Transonic, Ithaca, NY). A 1.5-mW green light laser (540 nm) (Melles Griot, Carlsbad, CA) was applied to the mid common carotid artery before injection of Rose Bengal (50 mg/kg in PBS) (Fisher, Fair Lawn, NJ) via tail vein. Arterial thrombosis was defined as flow cessation for at least 10 minutes. Flow in the carotid artery was monitored for 120 minutes.

### Platelet isolation and transfusion

Platelet isolation and transfusion were performed as reported previously^17^. Briefly, blood from the inferior vena cava was collected directly into 3.2% sodium citrate (9:1 blood/citrate ratio) and diluted with an equal volume of Tyrode’s buffer. Platelet-rich plasma (PRP) was obtained from whole blood by centrifugation at 50× g for 10 min. Then PRP was centrifuged at 1200 × g for 10 min, and the supernatant was discarded. The pellet containing platelets was resuspended in 500 μl PBS. Then platelet concentration was measured using a hemacytometer. For platelet transfusion experiments, suspended platelets from WT or *miR-223* ^−/*y*^ mice were diluted to a concentration of 1 × 10^8^ platelets in 150 ul PBS, and transfused via tail vein into recipient mice (WT or *miR-223* ^−/*y*^ mice). 4 hours after transfusion, carotid thrombosis was performed. Carotid arteries were collected after thrombosis study for further analysis.

### Platelet aggregation

Mice were anesthetized and PRP was collected as described above. Platelet concentration was measured using a hemacytometer and the concentration of platelets was corrected to 250 × 10^6^ platelets/ml with Tyrode’s buffer. ADP (10 μg/ml), collagen (5 μg/ml), or thrombin (0.1 u/ml)-stimulated platelet aggregation was performed using a Whole Blood/Optical Lumi-Aggregation System (Chrono-Log Corp., Havertown, PA, USA) according to the manufacturer’s instruction.

### Extracellular vesicle isolation and transfusion

To isolate extracellular vesicles (ECVs) released from platelets, the platelet pellet was resuspended in 1 ml PBS. Then 0.1 u thrombin was added, and platelets were incubated for 2 hours at 37 °C with agitation every 20 minutes. The PRP was centrifuged at 1200× g for 10 minutes. The supernatant containing ECVs was then centrifuged at 100,000× g for 2 hours at 4 °C. ECVs were collected from the pellet and resuspended in 150 μl PBS. ECVs (150 μl in PBS per mouse) were transfused via tail vein into recipient *miR-223* ^−/*y*^ mice. 4 hours after transfusion, carotid thrombosis was performed.

### Co-culture of ECVs and endothelial cells

Mouse brain endothelial cells (bEnd.3, ATCC, Manassas, VA) were grown in Dulbecco modified Eagle medium (DMEM, Gibco, Waltham, MA) containing 10% fetal bovine serum (FBS, Gibco, Waltham, MA). To study the transportation of miR-223 from ECVs to endothelial cells, bEnd.3 cells were seeded in 6-well plates. ECVs isolated from WT or *miR-223* ^−/*y*^ mice as above were added to bEnd.3 cells (500 μl/well). 12 hours after co-culture, the bEnd.3 cells were rinsed 3 times with PBS. Then bEnd.3 cells were collected for further analysis.

### Real-Time Polymerase Chain Reaction

RNA from the common carotid arteries was isolated using a QIAGEN RNeasy Mini Kit (QIAGEN Inc., Valencia, CA). The primer set for insulin growth factor-1 receptor (IGF-1R) was purchased from Applied Biosystems (Carlsbad, CA). For miR-223 expression, microRNA from bEnd.3 cells and the common carotid arteries was isolated using a mirVana miRNA isolation kit (Life Technologies, Fresno, CA). The primer sets for specific amplification were purchased from Applied Biosystems (Carlsbad, CA). RT-PCR was performed using an ABI Prism 7000 Sequence Detection System (Applied Biosystems, Carlsbad, CA). 100 ng of RNA and 1 μl of primers were used per reaction. 7000 System SDS Software and the 2^−ΔΔCT^ method^18^ were used to analyze the results. Results were presented as fold change of transcripts for target normalized to internal control (GAPDH or U6 snRNA)^19^.

### IGF-1R inhibition

A stock solution of selective IGF-1R inhibitor, AG-1024 (Cayman, Ann Arbor, MI), was prepared in DMSO at 5 mg/ml. At 10 weeks of age, WT or *miR-223* ^−/*y*^ mice were treated intraperitoneally with AG-1024 (2 mg/kg in PBS) overnight. Then carotid arterial thrombosis was performed to examine effect of IGF-1R inhibition.

### Statistical analysis

All data are presented as mean ± standard error. Statistical analysis was carried out using GraphPad Prism. Results were analyzed using unpaired t-test for comparisons between two groups. For multiple comparisons, results were analyzed using one-way ANOVA followed by Tukey post-test analysis. Probability values of p < 0.05 were considered statistically significant.
